# Comparative analysis of abdominal fluid cytokine levels in ovarian hyperstimulation syndrome (OHSS)

**DOI:** 10.1186/s13048-020-00624-9

**Published:** 2020-03-05

**Authors:** Balint Farkas, Ferenc Boldizsar, Noemi Bohonyi, Nelli Farkas, Saska Marczi, Gabor L. Kovacs, Jozsef Bodis, Miklos Koppan

**Affiliations:** 1 Department of Obstetrics and Gynecology, University of Pecs, School of Medicine, 17 Edesanyak Str., Pecs, Hungary; 2 Member of the HAS-UP Human Reproduction Scientific Research Group, Hungarian Academy of Sciences (HAS), Pecs, Hungary; 3 Department of Immunology and Biotechnology, University of Pecs, School of Medicine, Pecs, Hungary; 4 School of Medicine, Institute of Bioanalysis, University of Pecs, Pecs, Hungary; 5 Laboratory of Molecular and HLA Diagnostics, University Hospital Osijek, Clinical Institute of Transfusion Medicine, Osijek, Croatia; 6 Department of Medical, Chemistry, Biochemistry and Clinical Chemistry, University of Osijek, Faculty of Medicine, Osijek, Croatia; 7 Szentágothai Research Center, University of Pecs, Pecs, Hungary; 8 Department of Laboratory Medicine, Faculty of Medicine, University of Pecs, Pecs, Hungary

**Keywords:** Ovarian hyperstimulation syndrome, Ovulation induction therapy, Ovarian cancer, Ovarian endometriosis, Benign pelvic mass

## Abstract

**Background:**

Ovarian hyperstimulation syndrome (OHSS) is a rare, yet severe, iatrogenic complication of ovulation induction therapy during assisted reproductive procedures. Our group previously detected atypical cells in the ascitic fluid of OHSS patients, although no malignancy developed during follow up. Here, the aim was to perform a comparative analysis of the cytokines present in the abdominal fluid of patients affected by OHSS versus patients with advanced ovarian cancer, a benign adnexal mass, or ovarian endometriosis.

**Methods:**

This prospective, non-randomized study was conducted at the Clinical Center of the University of Pecs Department of Obstetrics and Gynecology/Reproductive Center between October 2016 and March 2018. Abdominal fluid samples were obtained from 76 patients and subjected to Luminex analysis. The samples were collected from patients with OHSS (OHSS; *n* = 16), advanced ovarian cancer (OC; *n* = 22), a benign adnexal mass (BAM; *n* = 21), or ovarian endometriosis (EM; *n* = 17). Data were subjected to the non-parametric Kruskal-Wallis test and Spearman’s rank correlation coefficient to identify statistical differences between the four study groups.

**Results:**

Leukocytosis and hemoconcentration were detected in the peripheral blood of OHSS patients. Abdominal fluid analysis further revealed significantly higher levels of interleukin (IL)-6, IL-8, IL-10, and transforming growth factor (TGF)-β in both the OHSS and OC groups compared to the BAM and EM groups. The highest concentration of vascular endothelial growth factor (VEGF) was detected in the OC group, while a significantly lower level was detected in the OHSS group. Moreover, VEGF levels in OC and OHSS groups were significantly elevated compared to the levels in the BAM and EM groups.

**Conclusions:**

Vasoactive and hematogenic cytokines were present at higher levels in both the OHSS and OC abdominal fluid samples compared to the fluid samples obtained from the peritoneal cavity of the BAM patients. It is possible that these cytokines play an important role in the formation of ascites.

## Background

Infertility is defined as an individual’s inability to reproduce through a natural process. Currently, infertility represents a major healthcare issue in the twenty-first century and it can be the result of male, female, or combined infertility issues. Worldwide, an estimated 48 million women and approximately 7% of men suffer from infertility [1, 2]. Thus, a growing need for assisted reproduction techniques, particularly in vitro fertilization (IVF) procedures, exists to facilitate conception. However, ovarian hyperstimulation syndrome (OHSS) is a rare, yet potentially life threatening, iatrogenic complication of ovarian induction therapy (OIT) during IVF procedures. OHSS is associated with abdominal pain and/or bloating, nausea, vomiting, and in severe cases, shortness of breath and chest pain. A diagnosis of OHSS is confirmed with laboratory findings of hemoconcentration and ultrasound imaging [3]. Manifestations of the disease can vary from mild to moderate to severe. OHSS often develops after the administration of gonadotropins which are needed to facilitate oocyte maturation and release during IVF procedures. The pathophysiology of OHSS is characterized by the appearance of multiple large luteinized cysts in the ovaries. These cysts are accompanied by a simultaneous increase in vascular permeability which leads to a shift in fluids from the intravascular system to the abdominal and pleural cavity [4].

Despite greater insights into the etiology of OHSS, the exact pathomechanism remains unclear. It has been hypothesized that local vasoactive mediators, such as vascular endothelial growth factor (VEGF), substances belonging to the renin-angiotensin system, and cytokines such as interleukin (IL)-6 and IL-8, play major roles in disease pathogenesis [5–9]. These factors can potentially induce fluid redistribution and massive extravasation, thereby resulting in a state of hypovolemic hyponatremia with hemoconcentration, as well as hypercoagulability [8, 9].

A growing concern among public opinion is a potential link between IVF procedures and malignant disease, and this issue may challenge the safety of assisted reproduction. To date, there is no clear evidence which demonstrates a causative role for IVF procedures in breast cancer [10, 11] or ovarian cancer [12]. However, we previously detected atypical cells in the ascitic fluid of women with severe OHSS [13]; although, no correlation between the presence of these cells and subsequent malignancy was observed [13]. Therefore, the aim of the current study was to analyze and compare the levels of potentially key mediators of OHSS in the ascitic fluid of women with OHSS, advanced ovarian cancer (OC), or ovarian endometriosis, and in the abdominal fluid of women with benign adnexal masses (BAMs). We hypothesize that these results will provide a better understanding of the pathomechanism of OHSS.

## Results

### Demographic characteristics

The mean age of our study groups were: 34 ± 5 years (range: 26–44) for the OHSS group; 64 ± 13 years (range: 30–84) for the OC group; 51 ± 15 years (range: 24–78) for the BAM group; and 34 ± 8 years (range: 18–47) for the ovarian endometriosis (EM) group.

### Peripheral blood serum analysis

**Fig. 1:**
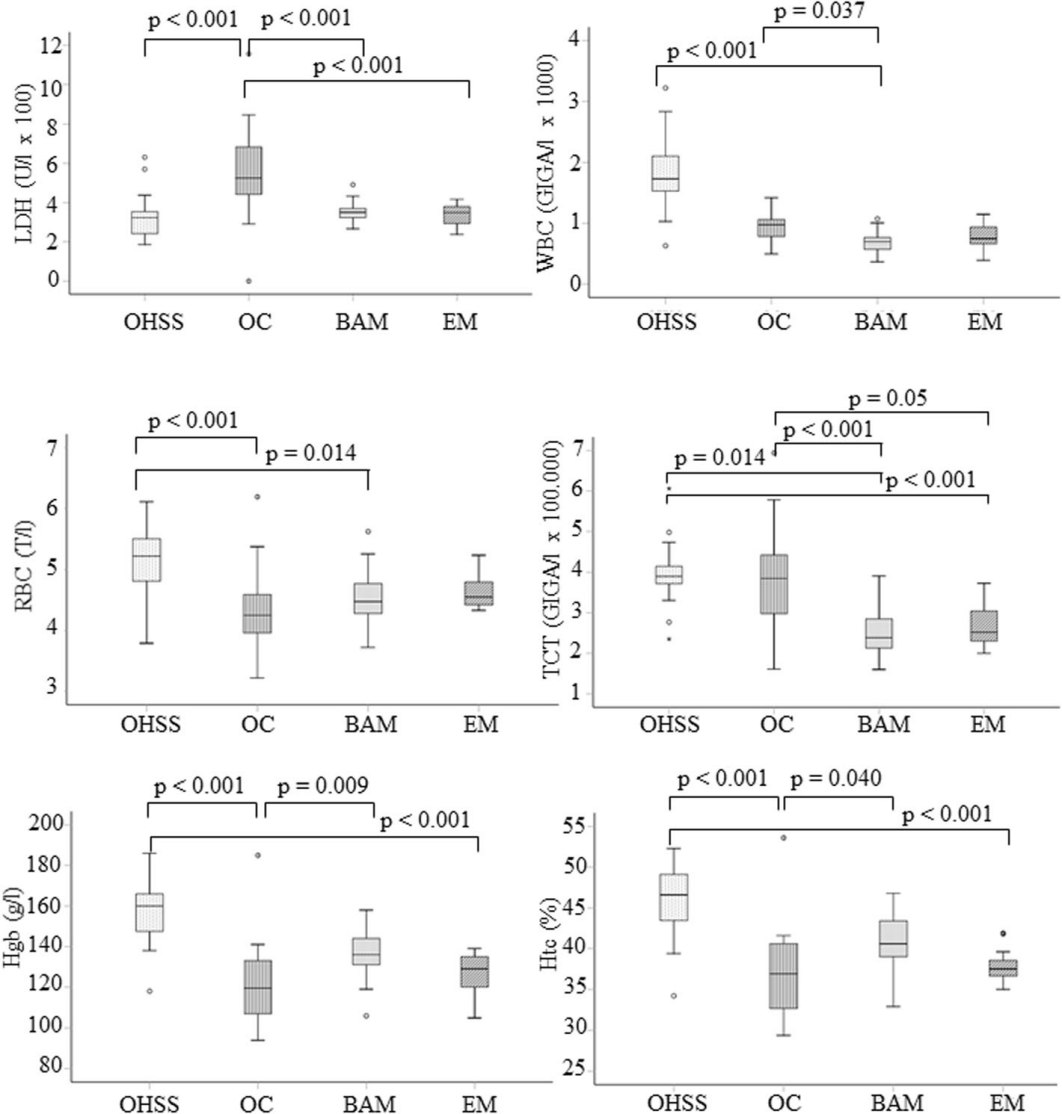
Levels of LDH and Hgb in peripheral blood serum and hematogram values for the four study groups. Comparison were made with non-parametric Kruskal-Wallis test with Bonferroni post hoc test

The mean serum levels of Na^+^ and K^+^, as well as activity levels for – aspartate transaminase (ASAT), alanine aminotransferase - (ALAT), and lactate dehydrogenase (LDH), are presented in Fig. 1. In addition, white and red blood cell counts (WBC and RBC, respectively), thrombocyte (TCT) count, blood hemoglobin concentration (Hgb), and hematocrit level (Htc) are also presented in Fig. 1. Application of the non-parametric Kruskal-Wallis test to these data revealed significant differences between the distribution of several values among the study groups (Fig. 1).

### Abdominal fluid cytokine level analysis

**Fig. 2:**
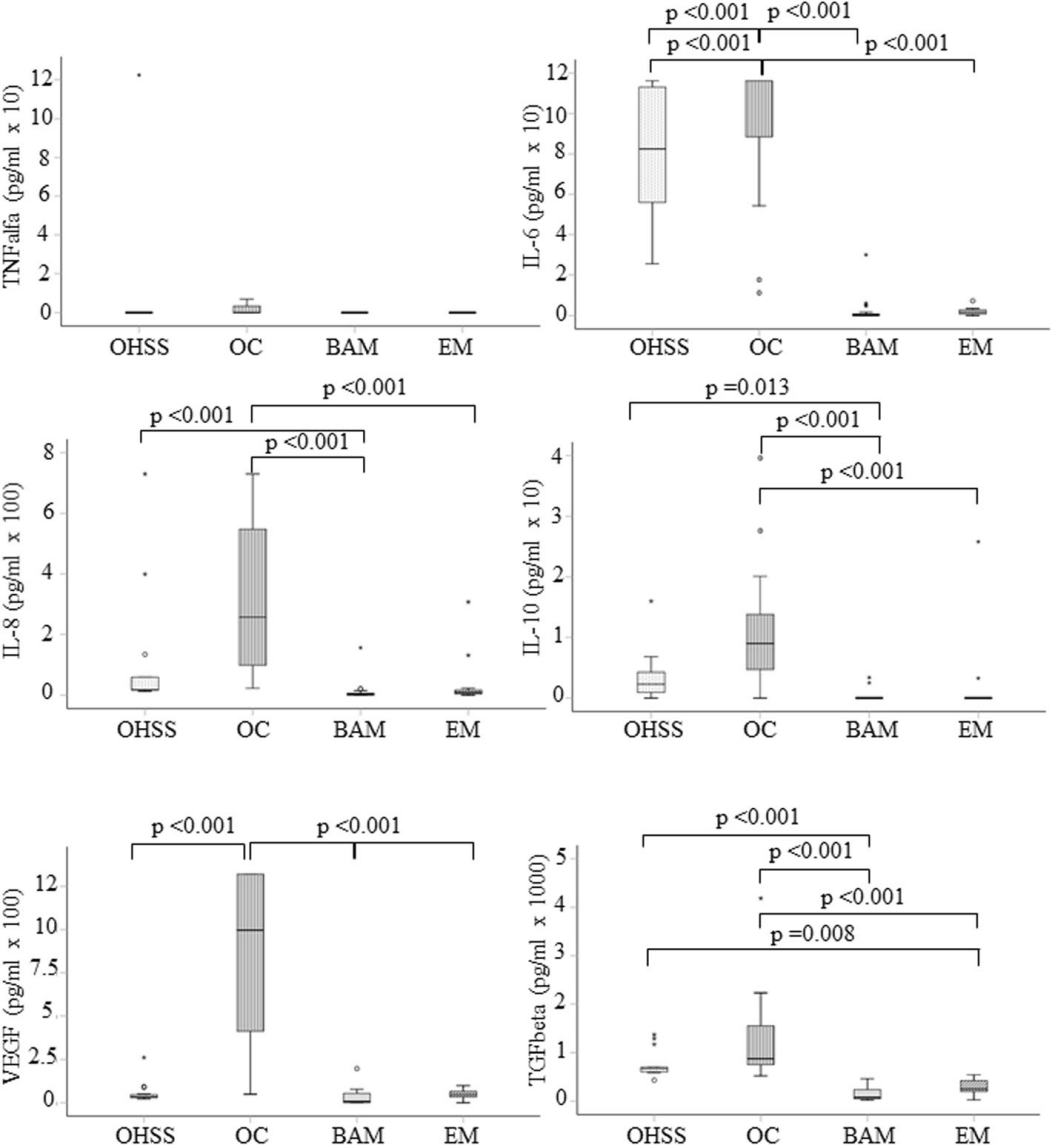
Cytokine levels in abdominal fluid samples obtained from the peritoneal cavity in the four study groups. Statistical analysis included non-parametric Kruskal-Wallis test with Bonferroni post hoc test

**Fig. 3:**
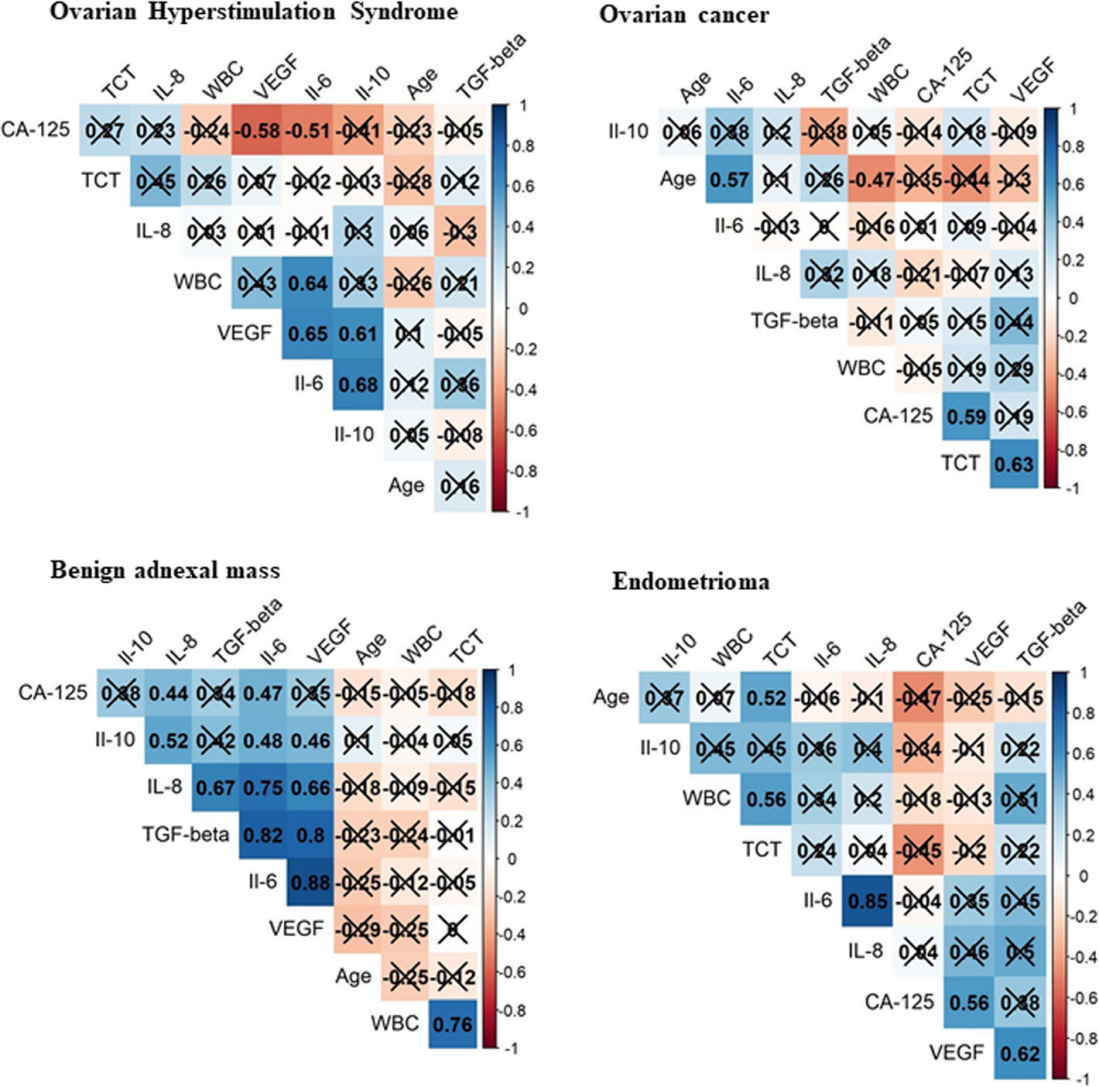
Spearman correlation matrix of the cytokine values, CA-125 tumor marker parameter and age. Correlation coefficients are shown, red in case of negative, blue in case of positive correlation. X marks: non significant connection

From the OHSS patients, an average of 1.1 l of ascites were withdrawn. In a Luminex assay, levels of six cytokines were investigated: IL-6, IL-8, IL-10, tumor necrosis factor (TNF)-α, VEGF, and transforming growth factor (TGF)-β. The mean concentration values for IL-6, IL-8, and TGF-β were significantly higher in both the OHSS and OC groups compared to the BAM and EM groups (Fig. 2). The level of VEGF was only significantly higher in the OC group. No statistically significant differences in TNF-α concentrations were observed among the four study groups (Fig. 2). With Spearman’s correlation analysis various significant positive correlations were observed in the OHSS group between the WBC count and IL-6 level (*r* = 0.640; *p* < 0.01), between the IL-6 and IL-10 levels (*r* = 0.677; *p* < 0.01), between the IL-6 and VEGF levels (*r* = 0.652; *p* < 0.01), and between the IL-10 and VEGF levels (*r* = 0.615; *p* < 0.01). In contrast, a significant negative correlation was observed between the serum CA-125 level and VEGF concentration in ascites (*r* = − 0.584; *p* < 0.01). Meanwhile, a significant positive correlation was observed between serum CA-125 level and abdominal fluid VEGF concentration in the EM group (*r* = 0.564; *p* = 0.02). In the OC group, a significant positive correlation between peripheral blood TCT level and serum CA-125 level (*r* = 0.568; *p* < 0.01), and between TCT and VEGF concentration (*r* = 0.624; *p* < 0.01), were observed. In the BAM group, the level of IL-6 in abdominal fluid exhibited a significant positive correlation with the levels of IL-8, IL-10, VEGF, and TGF-β. Similarly, IL-8 levels exhibited a significant positive correlation with the levels of IL-10, VEGF, and TGF-β, and also between the IL-10 level and the VEGF and TGF-β levels (See Fig. 3.)).

## Discussion

Cytokines are a group of polypeptides which are unable to penetrate the lipid bilayer of cells. Despite this limitation, it is still hypothesized that these peptides play an important role in cell signaling. In OHSS, roles for several cytokines have been well-established, thereby suggesting that interactions take place between the immune system and the ovaries during the development of this disease [14]. In the current pilot study, significant alterations in the levels of examined cytokines were observed in the abdominal fluid samples collected from our four study groups.

The pro-inflammatory cytokine, IL-6, is produced by various cells, including monocytes, T lymphocytes, endothelial cells, and fibroblasts [15]. It has also been proposed that IL-6 is a major mediator of ascites formation based on its involvement in angiogenesis and hyperpermeability [16]. The present results confirm that high levels of IL-6 are present in the peritoneal cavity of patients with severe OHSS and in patients with advanced ovarian cancer. In contrast, ascites was not detected in the BAM and EM patients. Recent studies also propose that increased serum levels and peritoneal cavity levels of both IL-6 and IL-10 are associated with factors of worse prognosis in ovarian cancer patients [6, 7].

Numerous studies have demonstrated that the vasoactive protein, VEGF, has a key role in OHSS. VEGF belongs to a family of heparin-binding proteins and is able to induce angiogenesis and vascular permeability [17, 18]. VEGF is secreted by ovarian granulosa cells and its production is stimulated by human chorionic gonadotropin hormone [4, 19–21]. Elevated levels of VEGF have been measured in both serum and ascitic fluid in patients with OHSS [21, 22]. These results, and those of the current study, are consistent with previous reports that high levels of VEGF are present in the ascitic fluid of patients with ovarian cancer [23] and also in the abdominal fluid of OHSS patients [24]. Meanwhile, low levels of VEGF were detected in BAM and EM patients in the present study. We hypothesize that increased production of VEGF is a major factor in the formation of ascites.

Macrophages produce IL-8, an important cytokine in the immune system. This cytokine induces chemotaxis-triggered neutrophil migration toward inflammation sites and then stimulates phagocytosis once the neutrophils arrive onsite [25]. Previously, levels of IL-8 and levels of the anti-inflammatory protein, IL-10, were found at higher concentrations in the ascitic fluid of OHSS patients [26, 27]. In our current investigation, significantly higher levels of both IL-8 and IL-10 were detected in the ascites of OHSS and OC patients compared to the levels detected in the abdominal lavage fluid of BAM and EM patients. Moreover, the levels were highest in the ascites of the OC patients. This finding is consistent with other recently published data [7] and with an angiogenetic role for IL-8 in malignancy [28] and a pivotal immunosuppressive role for IL-10 in OC-associated ascites when activation of dendritic cells via toll-like receptors is compromised [29].

To date, available literature does not indicate a consensus regarding the role of TNF-α in OHSS. For example, while no statistically significant difference was previously found in the amount of TNF-α in the ascites of OHSS patients compared to controls [30], others reported elevated levels in the same experimental setting [31]. The TNF-α data obtained in the present study support a less important role for TNF-α in OHSS.

TGF-β is a multifunctional cytokine. In its activated form, it binds TGF-β receptors by forming a serine/threonine kinase complex [31]. Subsequent activation of a signaling cascade leads to downstream activation of various substrates and regulatory proteins. In addition, the transcription of various target genes is induced, thereby contributing to differentiation, chemotaxis, proliferation, and activation of many immune cells [31]. In our study groups, a significant increase in the levels of TGF-β were detected in the OHSS and OC groups relative to the BAM and EM groups. The OC group had the highest concentration of TGF-β. Among the immunosuppressive cytokines associated with advanced ovarian cancer, it has been proposed that TGF-β contributes to impaired anti-tumor immune function [32]. However, the role of TGF-β in OHSS remains unknown.

The novelty of our data is that we managed to reveal similarly increased, with no statistically significant difference, in the peritoneal cavity levels of IL-6, IL-8, IL-10, VEGF and TGF-ß both in OC and OHSS patients, but found statistically significantly lower levels of the same cytokines compared to BAM and EM groups. Despite the mean age alteration between OC and OHSS groups the inflammatory responses might be hard to compare, but the cytokine production trend seem to be similar in these two groups. This might suggest same kind of pathomechanism of the ascites formation both in OHSS and in ovarian malignancy.

There are limitations associated with the present study. These include a relatively low number of participants, a lack of serum cytokine concentration measurements, discrepancies of age between the compared groups, which can influence the inflammatory profile, and some other, potentially important cytokine concentrations were not investigated as yet. Regarding the latter point, IL-2 would have been another cytokine of interest to investigate considering that it has been found at high levels in the peritoneal cavity of OHSS patients [33]. Furthermore, we could not isolate and identify the origin of the atypical cells present in the ascitic fluid of OHSS patients which we previously described [13, 34]. However, a strength of the present study is the broad spectrum of samples which were examined, including abdominal fluid from patients with various benign adnexal masses and from patients with ovarian “chocolate cysts” (e.g., endometrioma), which served as valid negative controls.

In the future a proposed potential clinical implication of our study would have been to find anti-inflammatory citokine agents to reduce the symptoms of OHSS, and to decrease the severity of the disease.

## Conclusions

Local pro- and anti-inflammatory cytokines, as well as vasoactive components, play important roles in both the formation of free abdominal fluid and in the pathogenesis of advanced ovarian cancer and OHSS compared with benign ovarian disease and ovarian manifestation of endometriosis. In further studies serum cytokine levels and peritoneal cavity immune cell distribution might worth to investigate, with the aim to reveal which cell population are colonize and produce the described cytokines.

## Methods

### Patients and study design

This prospective, non-randomized study was approved by the University of Pecs Institutional Ethical Review Board (#5273–2/2012) and was conducted at the Clinical Center of the University of Pecs Department of Obstetrics and Gynecology/Reproductive Center between October 2016 and March 2018. Patient participation was on a voluntary basis and all enrolled participants were older than 18 years of age. Written informed consent was completed if patients had an adnexal mass or if they were diagnosed with OHSS after OIT.

### Evaluation of abdominal fluid

Abdominal fluid samples were obtained during ultrasound-guided culdocentesis of patients with a severe form OHSS (*n* = 16), who represented the investigated population; intraoperative ascites sampling was performed during laparotomy of patients with advanced ovarian cancer (OC) (*n* = 22), who were the malignant disease group; sterile saline was collected after intraoperative pelvic lavage during laparoscopic cystectomy of patients with a benign adnexal mass (BAM; *n* = 21), who acted as negative controls; and intraoperative sampling of free abdominal fluid was performed during operative laparoscopy for patients with ovarian endometriosis (EM; *n* = 17), used as transient / benign disease group. Clinical and histological diagnoses of the participants are summarized in Table 1.

**Table 1:** Clinical and histological diagnoses of the participants in the four study groups

Study group	Histologic Diagnosis			Number (n)
OHSS	NA			16
Ovarian Cancer		FIGO Stage	Grade	22
	Serous papillary adenocarcinoma	3b-c	High	16
	Clear cell adenocarcinoma	NA	NA	2
	Adenosarcoma	NA	Low	2
	Adult granulosa cell ovarian tumor	3b	NA	1
	Borderline (atypical proliferation)	NA	NA	1
Benign adnexal mass				21
	Ovarian fibroma			6
	Follicular cyst			3
	Granulosa lutein cyst			6
	Adult type teratoma			1
	Borderline tumor (no atypical proliferation)			3
	Paraovarian cyst			2
Endometriosis	Endometrioma of the ovaries			17

*FIGO* International Federation of Obstetrics and Gynecology

### Peripheral blood analysis

Peripheral blood samples were collected preoperatively from all the study participants, including 60 patients who were admitted for surgery on the day of intervention, and samples were also collected on the day of hospitalization for the OHSS patients (*n* = 16). Serum levels of Na^+^, K^+^, LDH, ASAT, ALAT, and CA-125 tumor marker were determined. A hemogram was also performed.

### Measurement of cytokine levels in abdominal fluid

Cytokine levels were measured by using the R&D Systems Human Premixed Multi-Analyte Kit Luminex Assay (Cat. no. LXSAH-6; R&D Systems, Minneapolis, MN, USA) and a Luminex 200 instrument (R&D Systems). Levels of IL-6, IL-8, IL-10, TNF-α, and VEGF were measured according to the manufacturer’s instructions. TGF-β levels were measured with the R&D Systems Magnetic Luminex Performance Assay and MAGPIX MILLIPLEX MAP instrument (MilliporeSigma, Danvers, MA, USA) according to the manufacturer’s instructions. Samples above the standard curve were considered to be maximum value, and samples under the curve sensitivity were annotated as 0. All data are displayed in pg/ml.

### Statistical analysis

Statistical analyses were performed by using IBM SPSS Statistic 25 software (IBM Corporation) at the University of Pecs, Institute of Bioanalysis (performed by NF). The total sample size (n) was 76. Comparisons were made between serum and cytokine levels detected for the four study groups according to the non-parametric Kruskal-Wallis test with Bonferroni post hoc test. To examine the relationship between cytokine levels and serum parameters, Spearman’s rank correlation coefficient was applied. Mean data are reported ± standard deviation (SD). Statistical significance was set at *p < 0.05, or **p < 0.01.

## Data Availability

The datasets generated and/or analyzed in the current study are not publicly available in order to prevent compromise of individuals’ privacy. However, the data are available from the corresponding author upon reasonable request.

## A**bbreviations**

ALAT: Alanine Aminotransferase
ASAT: Aspartate transaminase
BAM: Benign adnexal mass
EM: Endometriosis
Hgb: Hemoglobin
Htc: Hematocrit, packed cell volume
IL: Interleukin
IVF: In vitro fertilization
LDH: Lactate dehydrogenase
OC: Ovarian cancer
OHSS: Ovarian hyperstimulation syndrome
OIT: Ovulation induction therapy
RBC: Red blood cell
SD: Standard deviation
TCT: Thrombocyte
TGF-β: Transforming growth factor-beta
TNF-α: Tumor necrosis factor-alpha
VEGF: Vascular endothelial growth factor
WBC: White blood cell
